# Dataset on comprehensive assessment & classification of upper & lower limb pain in athletes

**DOI:** 10.1016/j.dib.2024.110315

**Published:** 2024-03-19

**Authors:** Ciarán Purcell, Ciara Duignan, Brona Fullen, Shiofra Ryan, Tomás Ward, Brian Caulfield

**Affiliations:** a School of Public Health, Physiotherapy and Sports Science, University College Dublin, Dublin, Ireland; b Insight SFI Research Centre for Data Analytics, University College Dublin, Dublin, Ireland; c School of Allied Health, University of Limerick, Limerick, Ireland; d Physical Activity for Health Research Cluster, Health Research Institute, University of Limerick, Limerick, Ireland; e Sports and Human Performance Centre, University of Limerick, Limerick, Ireland; f Ageing Research Centre, Health Research Institute, University of Limerick, Limerick, Ireland; g Insight Centre for Data Analytics, School of Computing, Dublin City University, Dublin, Ireland

**Keywords:** Sport, Pain, Athletes, Assessment

## Abstract

Data were charted as part of a scoping review which followed the Joanna Briggs Institute (JBI) evidence synthesis guidelines and the Preferred Reporting Items for Systematic Reviews and Meta Analysis Scoping Review extension (PRISMA-SCr) guidelines. Data was extracted from 470 articles that met the inclusion criteria for the scoping review; primary research articles of athletes where upper and/or lower limb pain since database inception.

A draft data charting tool was developed by the research team and piloted for feasibility, accuracy and agreement. The charting tool was updated accordingly before being applied to the entire data set. Data collected included citation details, research context, participant information and pain assessment and classification tools, categories, and additional relevant information.

The raw data set was filtered, and descriptive analysis of frequencies and counts were conducted.

Researchers and clinicians interested in the range and applications of different pain assessment practices in athletes may reuse this data set. Data charting was comprehensive including aspects beyond the scope of the original research that offer clinical and research potential. These include information around recommended practice, (International Olympic Committee guidance) pain classifications and definitions and the use of multi-domain pain assessment tools.

Specifications Table

**Table:** 

Subject	Sport Sciences, Therapy and Medicine
Specific subject area	This data set addresses upper and lower limb pain assessment and classification tools and strategies in an athlete cohort extracted as part of a previously published scoping review [1]. Athletes are defined as being involved and competing regularly in a chosen sport [2]. The area spans local and amateur athletes to elite/professional athletes across all types of sports. Pain assessment tools include a wide range of measures and strategies to capture the sensory and emotional aspects of pain reflecting contemporary pain assessment strategies in a general population [3]. This data set includes studies where upper and lower limb pain (arms, legs, etc.) rather than spinal and central pain.
Type of data	Table Chart Graph Figure Excel Files
How the data were acquired	Relevant articles identified were imported into the Covidence reference management system (Covidence; Covidence Melbourne, Australia) where duplicates were removed. Abstract/title followed by full text review against the inclusion and exclusion criteria was completed by two independent members of the research team. 470 articles were identified for data charting. In accordance with the Johanna Briggs Institute (JBI) evidence synthesis methodological guidance, a draft data charting tool was developed and piloted by three independent members of the research team [4]. The charting tool was finalised in an iterative manner until a final tool was agreed for charting of the full data set previously published as an appendix [1].
Data format	Raw Analyzed (Descriptive Analysis) Filtered
Description of data collection	Data were taken directly from full texts of the included articles and reviewed in the Covidence reference manager software by the data charting team. (CP, CD, SR). Most data points were extracted from all 470 studies (where the data was reported). Three data points specific to case study designs were charted from the 190 case study design articles included in this study. Inclusion criteria were athletes (regularly competitive in their chosen sport) pain assessment (comprehensive pain assessment tools and strategies across both sensory and emotional aspects of pain) and upper and lower limb pain (pain in the arms and legs including muscle, bone, tendon, nerve and ligament pain but excluding pain in the torso or spine) [2]. Descriptions, criteria and examples of each of the data items are included as part of the published data set as an accompanying document titled “Data Labels & Descriptions” [5].
Data source location	The articles included in the scoping review and subsequent data set were identified from 6 databases; CINAHL (EBSCOhost), MEDLINE (PubMed) PsychINFO (ProQuest), EMBASE (Elsevier), SCOPUS (Elsevier) and the Cochrane Database of Systematic Reviews. The citation details for each of the 470 articles included are available in the data set. These include title, author, year of publication and DOI where available.
Data accessibility	Repository name: Open Science Framework (OSF) Data identification number: DOI 10.17605/OSF.IO/R64GX Direct URL to data: https://osf.io/r64gx/ All data collected is secondary data from previously published articles. Data is available for researchers to download directly via the OSF website link and requires Microsoft excel software to view. Data is freely available and the authors request that they be referenced when using, citing or sharing the dataset.

## 1 Value of the Data

• This is a unique data set of upper and lower limb comprehensive pain assessment and classification tools in athletes. The dataset includes a thorough set of variables around citation, article context, sporting setting and competition level, pain site and duration, athlete demographics and pain assessment data relating to the best available guidance: The International Olympic Committee Athlete Pain Framework [6].
• The depth and breadth of data included in this dataset was beyond that required for the scoping review and there is potential for further analysis including but not limited to; the pain assessment practice in case studies, (pain classification, diagnosis and onward referral) pain assessment in athletes with disabilities, pain classification and definition, multi domain pain assessment outcome measures, pain assessment practice in different sports (e.g. individual vs team sports) pain regions (e.g. shoulder, knee or ankle)and competition level.
• Will be of relevance and interest to researchers studying athlete pain across a range of demographic and contextual factors and clinicians seeking to understand how pain is assessed in different settings against best practice guidance
• There is a paucity of available data related to the assessment of upper and lower limb pain in athletes. This data set gives a comprehensive overview of all pain assessment tools used with athletes over a 50 year period 1971–2021. This dataset may be updated by future researchers to include data beyond 2021 to add to the body of knowledge.
• Researchers seeking to identify gaps for future studies where athlete pain is assessed can use this data set.

## 2 Objective

This comprehensive data set was gathered to give rich context and insight into the assessment and classification practices in upper and lower limb pain in athletes, an area which has not been explored in detail previously. The associated published article focuses on how pain assessment strategies map to the International Olympic Committee Athlete Pain Framework [6]. This data set provides rich contextual data and additional data related to best practice that was not discussed in the published article. There is value for clinicians and researchers alike in viewing the raw and analyzed data sets to understand assessment and classification practices in more detail. Additionally, there is added potential value from future secondary analysis.

## 3 Data Description

1. Raw data file. This Microsoft Excel file is the raw data extracted directly from Covidence using the data charting tool. Column data labels include; citation details, study details and context, participant demographics, pain and sporting context, pain assessment tools and categories and pain classification and definition information. Each row corresponds to an article.
2. Filtered and analyzed data set – Full data set tab. This Microsoft Excel sheet includes all the raw data alongside a small number of additional clearly labelled data columns used for summarizing and counting data. Filters of all data labels were applied to facilitate descriptive summaries and analysis. Row 474 and below contain the results of this descriptive analysis. The age data required an additional analysis and labelling step which is available to view in the “age data tab”
3. Filtered and analysed data set – Time trends tab. The time trends of pain assessment tools used over the previous 5 years (2017–2021) vs articles published prior to this date required an additional analysis step which is available to view in this tab. This tab includes 2 pie charts (Chart 1A & 1B) on the breakdown of pain assessment tools in each of the 5 pain assessment domains from 2017 to 2021 and prior to 2017. Additionally Figure 1 displays the pain assessment tools used across the 5 pain assessment domains over the past 50 years. This figure appears in the associated published article [1].
4. Filtered and analysed data set – Graphs & Tables Tab. This Microsoft Excel sheet consists of a series of tables and figures that summarise the data presented in the full data set tab. There are 4 figures and 18 tables, all of which are clearly labelled and correlate with the raw data and analysed data from the full data set tab.
5. Filtered and analysed data set – Filter tabs (Clinical, research and pain focused). Additional filters were applied to the dataset to compare trends across different categories; articles from a clinical context, articles from a research context and pain focused articles (containing a pain related aim, objective, or hypothesis). These filtered data sets are also presented in the graphs and tables tab; Papers from a clinical or service delivery context (green), research focused context (orange), pain focused (yellow) and papers published in the past 5 years (red).
6. Data Labels & Descriptions. Each of the column labels and data included are described in detail in this pdf document. This document also includes examples and further information around the analysis completed on the data set to familiarize readers with the data.

## 4 Experimental Design, Materials, and Methods

This data set was generated as part of a scoping review of the literature which followed the Joanna Briggs Institute (JBI) evidence synthesis guidelines and Preferred Reporting Items for Systematic Reviews and Meta Analysis extension for Scoping Reviews (PRISMA-SCr) guidelines [4,7]. The precise methods used were in keeping with a pre published protocol [2]. A comprehensive search of the literature was completed across 6 databases: CINAHL (EBSCOhost), MEDLINE (PubMed) PsychINFO (ProQuest), EMBASE (Elsevier), SCOPUS (Elsevier) and the Cochrane Database of Systematic Reviews. Relevant papers identified were imported into the Covidence reference management system (Covidence; Covidence Melbourne, Australia). Title and abstract followed by full text review screening against the inclusion and exclusion criteria was completed by two independent members of the research team. Data charting was completed by three members of the research team using the previously published charting tool [1]. (See supplementary material) Additional information around each of the data labels and the content included is available in the “Data Labels and Descriptions” (available at https://osf.io/gx2mk). After charting was completed, the data was exported from Covidence to Excel. The data file was scanned and cleaned by the lead researcher for any typographical/input errors. Headings were highlighted for readability. This Raw Data file is available at https://osf.io/d7y2r. The data set was then filtered, and descriptive analysis was performed by counting and summarizing each data label. This descriptive data was then used to generate a number of tables and graphs described above. The filtered and analyzed data set is available at https://osf.io/9nmqk.

## Ethics Statements

This data set was generated using previously published primary research. As this was secondary research ethical approval was not required for this study. This data set did not involve research conducted on humans or animals.

## CRediT authorship contribution statement

**Ciarán Purcell:** Conceptualization, Methodology, Validation, Investigation, Data curation, Writing – original draft, Writing – review & editing, Visualization. **Ciara Duignan:** Methodology, Validation, Investigation, Writing – review & editing. **Brona Fullen:** Methodology, Writing – review & editing. **Shiofra Ryan:** Validation, Investigation. **Tomás Ward:** Methodology, Writing – review & editing, Supervision. **Brian Caulfield:** Conceptualization, Methodology, Writing – review & editing, Supervision, Project administration.

## Data Availability

Dataset on Comprehensive Assessment & Classification of Upper & Lower Limb Pain in Athletes (Original data) (Open Science Framework (OSF)).

## Appendix Supplementary materials

Image, application 1

Image, application 2

Image, application 3
