# Supplementary material for: Dataset on comprehensive assessment & classification of upper & lower limb pain in athletes
Source: Data Brief. 2024 Mar 19;54:110315. doi: 10.1016/j.dib.2024.110315 (PMC11220924; doi:10.1016/j.dib.2024.110315)
Supplement: Supplementary file 1 [file mmc1.pdf]

Dataset labels & descriptions.
(Raw Data Set)

This data set was extracted from Covidence where a modified data charting template was used to extract the data. The file was exported to excel and some of the labels have been adjusted for clarity and reuse. This document describes the labels and data in each column.

Citation Details

Study ID
Author Surname and Year of Publication taken directly from Covidence reference management software.

Title
Full Title of Article as it appears on covidence

Study ID/ DOI/ PMID;
Where possible included DOI Eg 10.1016/j.arthro.2004.08.005
Included Pubmed ID, PMID or similar ID when DOI was unavailable.

Date (First Published online)
Year study was first published online

Study Details

Context
Researchers chose between a "research" context where the data captured was done so to explicitly answer a research question and a "clinical/service delivery context" where the data is part of routine data captured for clinical practice/normal service delivery.

1. <u>Research –</u>

Examples:

- RCT of physio treatments, supplement or soft tissue trial etc.
- Surgical trials with specific inclusion/exclusion criteria and protocols that differ from routine care were deemed research
- Studies pitch side or at a running event where additional data was captured such as V02/inflammatory markers that is not routine data capture is research context.

Investigating the effects of a recovery modality between groups was also deemed research.

2. Clinical/service delivery –

Examples:

- Reporting outcomes of normal post event recovery modalities. Reporting of pitchside/court side routine data collection. Reporting of routine surgical procedures and/or routine follow up.
- Includes retrospective review of charts/surgical outcomes.

Study Design Category

The category of research conducted was selected. Researchers chose from 4 broad study design categories
1. Case Studies.
2. Observational or epidemiological .
3. Experimental or Interventional
4. Qualitative or Mixed Methods.

1. Case studies include single or multiple case studies or retrospective reviews of multiple cases.

Examples
- Case study of a hamstring muscle tear or of a new acl graft procedure and relevant outcomes
- retrospective and prospective case studies and case series. Includes surgical studies of consecutive patients.

2. Observational/epidemiological studies including retrospective case controls, prospective cohort and cross sectional studies as well as population surveys and other forms of epidemiological studies.

Examples:

- Retrospective case control studies where groups with and without a specific variable of interest are compared eg those with/without hamstring strains.
- Prospective cohort studies where groups of participants are recruited and followed over time and exposure to certain variables are tracked alongside occurrence of outcomes of interest e.g strength training adherence is tracked over a season alongside those who sustain a hamstring strain and those who don’t.
- Cross sectional studies where a single point evaluation is completed and a set of data is collected e.g. a pre-season review of hamstring strength and flexibility measures of a team
- Cross sectional analytical (diagnostic studies where one technique is compared to another in the same group of participants e.g. a diagnostic accuracy study comparing the Ottawa ankle rules with X-ray/MRI for detection of bone fracture.

- Population surveys/epidemiological studies
- Eg. studies investigating the incidence and prevalence of hamstring strains in a team

3. <u>Experimental/Interventional</u> including RCTs, non randomised RCTs, experimental studies/trials.

Examples:

- -RCT where a treatment and control group are selected through randomisation and blinding is used. Eg. An RCT comparing the effects of a dry needling with sham dry needling where participants are randomised to each group
- Non/quasi randomised control trial where a treatment and control group are allocated through selection rather than randomisation either by the researchers/participants themselves.
- -Non controlled experimental trial where an intervention is carried out on a group of participants without a control group for comparison.
- Experimental studies where subjects are given a pain stimulus by heat probe etc and their response is monitored/compared is deemed an experimental/interventional study. IE. the design of the study is to determine the different pain response through experiment .

4. <u>Qualitative/Mixed Methods</u> including interviews, qualitative questionnaires, focus groups, user feedback and combinations of qualitative and quantitative methods.

Case Studies Additional Details

For the purpose of this study, 3 recommendations from the International Olympic Committee (IOC, 2017) for use when assessing athletes in practice were applied to all research of case study design that we identified. Case studies were deemed a reflection of clinical practice. The research team were interested in whether the case studies included 1) A diagnosis 2) The pain mechanisms involved and 3) Need or consideration of onward referral to another healthcare practitioner, consultant physician or for diagnostic imaging.

Diagnosis

Did the study give a specific diagnosis/pathology. This may include a specific structural or functional diagnosis +/- how that diagnosis was established.
Eg. a grade 1 hip flexor strain, disc related back pain, grade 1 ATFP sprain.

Pain Mechanism

Did the study explicitly assess or discuss the predominant pain mechanism under the categories of the International Association for the Study of Pain (IASP) which are detailed below. They may clearly state the pain mechanism or describe an assessment process to rule out neuropathic pain such as use of neurological pain screening questionnaires or neurological screening (Full neuro exam) but neuropathic pain must be mentioned

-Nociceptive, routine tissue injury pain (pain that arises from actual or threatened damage to non-neural tissue and is due to the activation of nociceptors.) May include inflammatory pain as a subcategory of nociceptive
-Neuropathic (pain that arises as a direct consequence of a lesion or diseases affecting the somatosensory system)- eg spinal cord injury, diabetic neuropathy.
-Nociplastic (Pain that arises from altered nociception despite no clear evidence of actual or threatened tissue damage causing the activation of peripheral nociceptors or evidence for disease or lesion of the somatosensory system causing the pain.) More prevalent in chronic pain presentations.

Referral

Did the study discuss the potential need for onward referral to another clinician or for diagnostic imaging. This may involve discussion around medical/clinical history, screening of red flags/serious pathology etc. This may include referral for imaging such x-ray/MRI to outrule other pathologies

Pain Related aims.

Study which included an athlete pain related aim hypothesiss or objective.
Researchers identified studies where authors set out with a research question related to pain/athlete pain. Aims related to injuries/pathology only were not included. Where aims describe a set of measures such as physiological, neuromuscular etc and some of those measures are pain measures these are also included

Example

- this study aims to assess the experience of patellofemoral pain in athletes or the pressure pain threshold/pain tolerance levels in endurance athletes etc
- Study investigating neuromuscular recovery measures in lower limb post soccer game and one of the measures is leg pain.
- Aims/objectives/hypothesis around delayed onset muscle soreness were included.

Aims Text / Limitations Text

Researchers copied text from original article where a pain related aim, hypothesis or objective was identified or where limitations/future directions was identified respectively.

Pain related Limitations/Future Directions.

Limitations/Future directions discussing or related to athlete pain concepts, ideas or challenges Studies discussing future directions of pain research, the difficulties of assessing pain in athletes or theories, concepts ideas related to pain mechanisms, presentations, experiences etc. Limitations were taken only from specific limitations sections or paragraphs where limitations were clearly articulated. Future directions also included recommendations for clinical practice around pain or pain conditions.

Example

- a suprapatellar plica should be outruled as potential cause of knee pain

Participants

Male N=, Female N= Not Stated N=

Total number of male/female/not stated athlete participants included. Where possible efforts were made to extract just the participant numbers which were from athlete cohorts. I.E for studies which included cohorts of participants which were not athletes as well as athletes only the athlete cohort were counted.

Age(Male), Age(Female) Age(combined)
Age related data was extracted as it was reported in the study. Where relevant we included mean, standard deviation, and range. Where appropriate we entered the information under male, female and combined categories.

Only age data where the information for the specific athlete cohort was provided was entered. Data that described the entire study cohort but not the specific athlete cohort was therefore not charted.

Sport
All sports that applied in each study were selected and charted. Athletics was divided into two categories. Track/running encompasses all running events from short sprints, to middle distance to long distance on track, trail, road or cross country. Field encompasses all throwing/jumping events.

Competition level

A comprehensive list of competition levels as outlined below was used. Where studies reported athletes from more than one level or the competition level was unclear mixed was selected.

Local/club/amateur - includes most entry level athletes. This included studies around events where specific levels were not identified and no other categories were relevant. Also included intramural sports (college club sports/5-a-sides etc)

Primary school - in general athletes 0-12 years old, for the most part this included little league baseball etc.

High school/secondary school in general 13-18 and involves all post primary competition levels and academy athletes in some cases.

Collegiate - includes all competition levels ie Division 1 division 5, intervarsity etc.

Semi Professional - indicates some payment/financial reimbursement but not as their full time occupation

Professional/international/elite - includes all full time occupation athletes, all national team squads etc and high performing/elite athletes - top tier of competition.

Mixed was selected in all settings where athletes do not all fall into one specific category.

Paratheltes

A separate question to indicate inclusion of parathletes was included. This included wheelchair sports amongst others.

Athletes with Intellectual Disability

A separate question to indicate inclusion of athletes with intellectual disabilities was included.

Pain Region

Upper limb or lower limb pain was charted. Mixed was select where athletes in the study had pain/pain assessed in both upper and lower limb regions.

Upper Limb Pain Regions & Lower Pain Limb Regions

All pain regions/areas that applied were charted. Regions selected were those related to pain only. Where regions related to strength or sensory loss but not pain were described these were not included.

Time Based Pain Classifications

In the first instance we recorded author defined timelines/duration (some definitions of acute, sub-acute and chronic vary)

Where the author did not explicitly state acute, sub-acute or chronic the below definitions Were used

acute pain 0-6 weeks,
subacute 6-12 weeks
chronic 12weeks+.

Pain Assessment Tools & Categories

All categories and tools that applied to those identified in the study were charted. Each pain assessment tool charted was matched to a pain assessment category. Individual names of each pain assessment tool were not chartered rather the most appropriate label/labels were selected. Where the charting form did not have an appropriate label the charting form was updated in an iterative fashion to insure all possible pain tools and categories were included. Pain tools were charted from inclusion/exclusion criteria, participants, and methods sections.

Trans/Multi domain Patient reported outcome measures/psychometric tools.

This question addressed whether a multi domain tool was used or identified.

Eg – Victorian Institute f Sport Assessment – Achilles (VISA A) Knee Injury & Osteoarthritis Outcome Score (KOOS), Hip & Groin Outcome Score (HAGOS) and other tools that may span multiple domains, categories, or tool headings were charted.

Note* - These measures often included pain assessments across multiple pain assessment tools and categories. All categories and pain assessment tools that could be identified from each multidomain tool was therefore charted.

List the Multi domain /PROMS used

The names of the tools used were charted

Pain Classification & Definition

Pain classification system or model?

This recorded whether authors reported a pain classification system or model.

This was identified either explicitly through the authors stating a specific classification system used or implicitly where we identified specific terminology and descriptions relating to a classification system or model

List the pain classification model/system.

A summary of the pain classification systems included is provided below.

The IASP pain classification system has been used widely and been established as the standard of practice in pain. Categories include nociceptive, neuropathic, nociplastic, and inflammatory.

The Biopsychosocial classification system is also commonly used in healthcare and may include categories or dimensions such as biomechanical, psychosocial, environmental, sociocultural and socioenvironmental.

Traditionally a biomedical model would have been prevalent with specific tissues or structures or even “pain receptors” being identified as the dominant pain “driver”.

Structural classifications of pain were treated as biomedical pain classification models. Where the author clearly attributes pain mechanism to a tissue or structure the biomedical pain classification category was selected.

The neuromatrix model was a follow up to the biomedical model as pain neuroscience began to develop and the role of the brain in pain perception was emphasised. Here we see a shift to an emphasis on specific pain regions such as the sensory, affective and cognitive brain regions. It differs to more modern approaches as the interaction between brain, body, environment etc is absent in this classification system.

Somatosensory profiling is largely based on aspects such as levels of sensitisation, conditioned pain modulation and other quantitative sensory measures. Somatosensory receptors include mechanoreceptors, thermoreceptors, proprioceptors, and chemoreceptors.

The ICF or International Classification of Functioning conceptualises a person's level of functioning as a dynamic interaction between her or his health conditions, environmental factors, and personal factors. It is a biopsychosocial model of disability, based on an integration of the social and medical models of disability. The ICF is broken down into functioning and disability and contextual factors. The first part incorporates body functions/structural impairments, activity limitations and participation restrictions. Contextual factors include personal and environmental factors.

Pain Definition

Pain definition used?
Yes was selected where the authors clearly defined pain. Only definitions of the term pain (rather than different types of pain) were included.

Quote Definition Below
We then extracted the definition directly from the text.

Pain definition label

Where specific organisation definitions such as the International Association for the Study of Pain (IASP) or the International Olympic Committee (IOC) definitions were used these were labelled.

References:

1. Hainline B, Derman W, Vernec A, et al. International Olympic Committee consensus statement on pain management in elite athletes. *Br J Sports Med* 2017;51(17):1245-58. doi: 10.1136/bjsports-2017-097884 [published Online First: 2017/08/23]

2. King W. (2013) Acute Pain, Subacute Pain, and Chronic Pain. In: Gebhart G.F., Schmidt R.F. (eds) Encyclopedia of Pain. Springer, Berlin, Heidelberg. https://doi.org/10.1007/978-3-642-28753-4_75Chronic >3months - ICD -11 Chronic Pain Definition

3. Marin, T.J., Van Eerd, D., Irvin, E., Couban, R., Koes, B.W., Malmivaara, A., Van Tulder, M.W., Kamper, S.J., 2017. Multidisciplinary biopsychosocial rehabilitation for subacute low back pain. Cochrane Database of Systematic Reviews 2017.. https://doi.org/10.1002/14651858.cd002193.pub2

Dataset labels & descriptions.
(Filtered & Analysed Data Set)

Additional Columns/Labels on Full Data Set tab

Number of Neurophysiological, Biomechanical, Affective, Socioenvironmental and Cognitive categories.

This column was added to count the number of pain categories addressed in each paper. The results are summarised in the totals section below.

Counts

These columns were added to facilitate counting total number of pain assessment tools and used in each domain.

Data Frequencies, summaries and totals

Descriptive analysis was completed and is presented from row 472 downwards. Filters were applied to the data set across each of the columns to facilitate this descriptive analysis.

Data for the diagnosis, pain mechanism and referral questions are provided for the case study design articles (n=190) only as these were deemed a reflection of clinical practice.

All other data is charted from the entire data set of 470 articles.

Age Data

Age category frequencies required an extra analysis step. This step can be seen in the "age data' tab of the excel file. All raw data was labelled under the following headings.
Paediatrics (0-12)
Adolescent (13-17)
Adult (18-64)
Elderly (65+)

Where only mean and standard deviations of ages of participants were reported the appropriate label was estimated by the lead author.

Graphs & Tables tab

Data was taken from the data frequencies, summaries and totals section which is discussed above and visualised through tables and graphs. For the most part data included in blue graphs and tables are from the entire data set.

Additionally we completed some additional filters of the data set to compare trends across different context. These are included in different colours in the tables and graphs Namely:

- Papers from a clinical or service delivery context (green) vs research focused context (orange)
- Pain focused or papers which included a pain related aim, hypothesis or objective vs those that did not. (yellow)
- Papers published in the past 5 years vs papers published prior to the last 5 years (red) these data can also be seen under the “time trends” tab

Throughout the graphs and tables tab we highlighted findings that differed form the main data set that may be interesting in red text.
